# Bovine blood derived macrophages are unable to control *Coxiella burnetii* replication under hypoxic conditions

**DOI:** 10.3389/fimmu.2023.960927

**Published:** 2023-01-30

**Authors:** Michael Mauermeir, Martha Ölke, Inaya Hayek, Jan Schulze-Luehrmann, Katja Dettmer, Peter J. Oefner, Christian Berens, Christian Menge, Anja Lührmann

**Affiliations:** ^1^ Mikrobiologisches Institut, Universitätsklinikum Erlangen, Friedrich-Alexander-Universität Erlangen-Nürnberg, Erlangen, Germany; ^2^ Institut für Funktionelle Genomik, Universität Regensburg, Regensburg, Germany; ^3^ Friedrich-Loeffler-Institut, Institut für molekulare Pathogenese, Jena, Germany

**Keywords:** *Coxiella burnetii*, bovine macrophages, normoxia, hypoxia, HIF1α, STAT3, citrate, TNF

## Abstract

**Background:**

*Coxiella burnetii* is a zoonotic pathogen, infecting humans, livestock, pets, birds and ticks. Domestic ruminants such as cattle, sheep, and goats are the main reservoir and major cause of human infection. Infected ruminants are usually asymptomatic, while in humans infection can cause significant disease. Human and bovine macrophages differ in their permissiveness for *C. burnetii* strains from different host species and of various genotypes and their subsequent host cell response, but the underlying mechanism(s) at the cellular level are unknown.

**Methods:**

*C. burnetii* infected primary human and bovine macrophages under normoxic and hypoxic conditions were analyzed for (i) bacterial replication by CFU counts and immunofluorescence; (ii) immune regulators by westernblot and qRT-PCR; cytokines by ELISA; and metabolites by gas chromatography-mass spectrometry (GC-MS).

**Results:**

Here, we confirmed that peripheral blood-derived human macrophages prevent *C. burnetii* replication under oxygen-limiting conditions. In contrast, oxygen content had no influence on *C. burnetii* replication in bovine peripheral blood-derived macrophages. In hypoxic infected bovine macrophages, STAT3 is activated, even though HIF1α is stabilized, which otherwise prevents STAT3 activation in human macrophages. In addition, the TNFα mRNA level is higher in hypoxic than normoxic human macrophages, which correlates with increased secretion of TNFα and control of *C. burnetii* replication. In contrast, oxygen limitation does not impact TNFα mRNA levels in *C. burnetii*-infected bovine macrophages and secretion of TNFα is blocked. As TNFα is also involved in the control of *C. burnetii* replication in bovine macrophages, this cytokine is important for cell autonomous control and its absence is partially responsible for the ability of *C. burnetii* to replicate in hypoxic bovine macrophages. Further unveiling the molecular basis of macrophage-mediated control of *C. burnetii* replication might be the first step towards the development of host directed intervention measures to mitigate the health burden of this zoonotic agent.

## Introduction

*Coxiella burnetii* is an obligate intracellular pathogen, which causes the zoonotic disease Q fever. In humans, Q fever might be asymptomatic or presents as a mild self-limiting flu-like disease. However, the infection can also progress to an interstitial pneumonia or hepatitis (1). The severity of the primary infection does not predict long-term health consequences, such as post-Q fever fatigue syndrome (QFS) or chronic Q fever (2, 3). QFS occurs in ~20% of patients with symptomatic acute Q fever (4). QFS is defined as fatigue lasting longer than six months in combination with pain, sleeping problems, headache, and concentration issues. Importantly, there is no evidence-based treatment available (5). Approximately 2% of Q fever patients might develop chronic Q fever (6). Endocarditis is the most common manifestation of chronic Q fever, and patients with an underlying valvulopathy are at a higher risk (7). Treatment of chronic Q fever involves administration of doxycycline and hydroxychloroquine for 18-24 months (6).

The primary reservoir of *C. burnetii* and the major source of human infections are infected livestock (8). Infection in cattle, goats, and sheep is often asymptomatic, but it may also lead to metritis, infertility, abortion, stillbirth or the delivery of weak offspring (9). Infected females shed the pathogen in huge quantities through birthing products and in smaller numbers in urine, feces and milk. However, the route of *C. burnetii* shedding seems to differ between cattle, sheep, and goats. Thus, shedding in milk is common in cattle, but less widespread in ewes (8). Infection of humans occurs mainly *via* the respiratory tract, while ingestion of contaminated milk plays a lesser role in transmission of *C. burnetii* (1, 8). Most human Q fever outbreaks are linked to infected sheep and goats (10), though cattle may also serve as a source for human infections (11). Given a prevalence of ~30% of *C. burnetii* infection in cattle (12–16), we have to increase our understanding of how *C. burnetii* adapts to the bovine host *in vivo* and *in vitro*.

*In vitro* studies using bovine cells are rare, but demonstrate that *C. burnetii* infects and replicates inside bovine epithelial cell lines as well as in primary bovine monocyte-derived macrophages. However, *C. burnetii* only induces a transient pro-inflammatory cytokine response in macrophages. Reduced levels of pro-inflammatory cytokines correlate with bacterial replication, suggesting that intracellular *C. burnetii* replication depends on a suitable microenvironment (17–19). This is in agreement with previous findings, showing that TNF inhibits *C. burnetii* replication (20). Not only the cytokine profile at the site of infection is determinative for bacterial replication, but also the metabolism of the host (21). The availability of the TCA cycle metabolite citrate is essential for *C. burnetii* replication in human and murine macrophages (22). Under oxygen limiting conditions, the transcription factor hypoxia-inducible factor (HIF) 1α is stabilized (23). HIF1α limits the activation of STAT3, which in turn reduces the intracellular level of citrate and, as a consequence, *C. burnetii* replication (22). HIF1α does not only influence host cell metabolism, but also inflammation as it controls the expression and secretion of the pro-inflammatory cytokine TNF (24).

In this study, we aimed to investigate the intracellular *C. burnetii*-containing vacuole (CCV) in bovine cells in more detail, and whether oxygen availability influences host cell metabolism and immune response during *C. burnetii* infection in bovine macrophages.

## Material and methods

### Reagents and bacterial strain

Chemicals were purchased from Sigma Aldrich unless indicated otherwise. *C. burnetii* Nine Mile phase II clone 4 (RSA439), kindly provided by Matteo Bonazzi (CNRS, Montpellier, France), was grown in ACCM-D medium (Sunrise Science Products) at 37°C, 5% CO_2_ and 2.5% O_2_ for 7 days.

### Bovine peripheral blood derived macrophages

Bovine peripheral blood samples were obtained from healthy heifers of the Holstein-Friesian breed, kept at the Friedrich-Loeffler-Institut, Jena. The animal experiment was reviewed by the Committee on the Ethics of Animal Experiments and the Protection of Animals of the State of Thuringia, Germany, and approved by the competent authority (Permit numbers 22-2684-04-04-102/15, date of permission 17.06.2015, and 22-2684-04-BFI-20-102, date of permission 20.04.2020). Bovine macrophages from peripheral blood were isolated as described (19). Briefly, leukocytes were isolated from citrate whole-blood (5:1 dilution with a 3.8% sodium citrate solution) *via* centrifugation (2,380 x g, 20 min, room temperature), washed 3 times with PBS/EDTA (0.8% NaCl, 0.02% KH_2_PO_4_, 0.02% KCl, 0.142% Na_2_HPO_4_ x 2H_2_O, 0.2% Na-EDTA x 2H_2_O in ddH_2_O, pH 7.4) by centrifugation (800 x g, 10 min, room temperature). This was followed by an erythrocyte lysis step in lysis buffer (0.826% NH_4_Cl, 0.109% NaHCO_3_, 0.0037% Na_2_-EDTA x 2H_2_O in ddH_2_O) for 5 min. After three more washing steps with PBS/EDTA (300 x g, 10 min), the cells were layered onto Pancoll (Pan Biotech) for density centrifugation (800 x g, 45 min). The mononuclear cell layer was extracted and washed several times with 0.9% NaCl before incubation in Saint-Gobain VueLife^®^ “C” Series Bags (32-C) in Iscove´s modified Dulbecco´s medium (IMDM without phenol red, supplemented with 0.05 µM 2-mercaptoethanol, 20% FCS and antibiotics (1% Penicillin/Streptomycin, 1% Amphotericin B).

After 7 to 9 days of incubation at 37°C and 5% CO_2_, the cells were re-isolated from VueLife bags, washed several times with cold 0.9% NaCl solution (300 x g, 10 min, room temperature) and resuspended in IMDM medium without phenol red, supplemented with 0.05 µM 2-mercaptoethanol, 2% FCS and antibiotics (1% Penicillin/Streptomycin, 1% Amphotericin B). The cells were seeded into suspension culture plates. After 20-24 h incubation, non-adherent cells were washed away *via* gentle flushing with medium or sterile 0.9% NaCl solution. Cells were replenished with IMDM medium without phenol red, supplemented with 2-mercaptoethanol, 4% FCS (w/o antibiotics) and incubated at 37°C and 5% CO_2_ before further processing.

### Human peripheral blood derived macrophages

Human peripheral blood samples were obtained from healthy donors at the University Clinic of Erlangen (Ethical Committee Erlangen approval number 111_12B). Peripheral blood mononuclear cells (PBMCs) were harvested from Leukoreduction system chambers using a Pancoll-gradient centrifugation protocol as described earlier (22). In brief, Leukoreduction system chamber content was diluted with PBS (Merck) and layered onto Pancoll (Pan Biotech) for density centrifugation (1,328 x g, 25 min, room temperature). The leukocytes were collected and the remaining erythrocytes lysed *via* a brief incubation in cold ddH_2_O. After washing in PBS, PBMCs were positively selected using anti-CD14 beads (Miltenyi Biotec) according to the manufacturer’s protocol. The selected cells were cultivated for seven days in cRPMI (complete RPMI, RPMI 1640 containing 10% FCS, 1% HEPES, 0.5% 2-mercaptoethanol, 1% Penicillin/Streptomycin and 5µl/ml human M-CSF [≙ 50 U/ml, Peprotech]) in cell culture flasks. After seven days, cells were detached with Accutase/PBS (1:4) for 30 min at 37°C and gentle rinsing with PBS, before they were harvested by centrifugation (300 x g, 5 min, room temperature), resuspended in cRPMI w/o antibiotics, and seeded into cell culture plates. The cells were incubated at 37°C and 5% CO_2_ until further processing.

### Infection of cell lines with *C. burnetii*

Bel-26 and A549 cells were seeded and cultured in DMEM, low glucose and pyruvate (Thermo Fisher), containing 10% FCS and 5% FCS, respectively. The cells were incubated at 37°C, 5% CO_2_, 21% O_2_. For infection, *C. burnetii* was quantified *via* OD_600_ measurement (OD_600_ 1 ≙ 10^9^ C*. burnetii/*ml). The cells were infected at MOI 200. After 2h of infection, cells were washed 3 times with PBS to remove residual bacteria in the medium followed by the addition of fresh medium.

Bovine or human macrophages were infected at MOI 10 and incubated either under normoxic (N – 37°C, 5% CO_2_, 21% O_2_) or hypoxic (H – 37°C, 5% CO_2_, 0.5% O_2_) conditions.

### Quantitation of human and bovine TNFα

Secretion of TNF into cell culture supernatants by human or bovine macrophages either treated with 10µg/ml LPS from *E. coli* O111:B4 (Sigma) for 4 and 24 h or infected with *C. burnetii* for 4, 24 and 96 h were measured by ELISA (BD Biosciences or R&D Systems respectively). Additionally, TNFα secretion by bovine macrophages was determined by a bioassay deploying PK-15 cells as previously described (25, 26).

### Volume measurement of the CCVs

Z-stack images were taken using the LSM700 (Zeiss) as described before (27). The longest distance of the CCV and the corresponding 90 degree angle were measured with the Zen software (Zeiss). The XYZ dimensions of each CCV were multiplied and plotted as volume with GraphPad Prism 9 (GraphPad software, San Diego, USA).

### NAD^+^/NADH assay

Bovine and human macrophages were seeded at a density of 2x10^6^ cells per well on a 6-well plate. One day after seeding the cells were either left uninfected or were infected with *C. burnetii* at MOI 10 and incubated under normoxia or hypoxia. At 24 h post-infection, the levels of NAD^+^ and NADH were measured using the colorimetric NAD/NADH Assay Kit (Abcam) as described in the manufacturer´s protocol.

### Phagocytosis assay

Bovine and human macrophages were seeded at a density of 5x10^5^ cells per well on a 24-well plate. One day after seeding, cells were incubated with pHrodo Red *E. coli* bioparticles in accordance with the instruction (Thermo Fisher Scientific) for 30 min. The cells were washed with PBS, fixed for 20 min with 4% paraformaldehyde (Alfa Aesar) in PBS (Merck) and permeabilized for 2 min with ice-cold methanol. The samples were mounted onto glass slides using ProLong™ Diamond Antifade Mountant with DAPI (Thermo Fisher Scientific) to stain DNA. Sample analysis was performed using a Zeiss LSM 700 confocal microscope.

### Measurement of cellular metabolites by GC-MS

Bovine and human macrophages were seeded at a density of 10^7^ cells per 10-cm plate and infected with *C. burnetii* at MOI 10. Infected cells were pelleted at the time points indicated. Cells were then lysed and extracted for carboxylic acid analysis by GC-MS as previously described in detail (22, 28).

### Colony forming units

The supernatant of infected cells was removed. The adherent cells were incubated with ice-cold ddH_2_O for 10 min at room temperature and 30 min at 4°C. Next, the cells were ruptured *via* repeated pipetting (~50 x), the lysate was transferred to 1.5 ml Eppendorf tubes and pelleted at 21,000 x g for 1 min. The pellets were resuspended in ACCM-D medium and frozen at -20°C until further processing.

The thawed samples were briefly mixed and each sample was plated as triplicates onto ACCM-D agar plates in 10-fold dilution series. Inoculated plates were incubated for 14 days before counting the colony forming units.

### RNA

After removal of the cell culture supernatant, 500 µl of TriFast (Peqlab) or RNA-solv (VWR) were added to the dish. Cells were detached *via* gentle pipetting and transferred to RNAse-free microcentrifuge tubes. The tubes were vortexed for 15 s at maximum speed and stored at -80°C until RNA extraction. RNA extraction was performed according to the manufacturer’s protocol (Peqgold TriFast™ or VWR RNA-Solv^®^). To maximize RNA yield, 2 µl of Pelletpaint (Merck Millipore) or 1.5 µl Glycoblue coprecipitant (Thermo Fisher) were added to the samples in the isopropanol precipitation step. A switch to RNA-solv and Glycoblue coprecipitant was necessary due to a shortage of supply and discontinuation of TriFast.

### qRT-PCR

Reverse transcription of RNA to cDNA was performed using SuperScript™ II Reverse Transcriptase kit with oligo(dT)12-18 primers (Thermo Fisher) according to the manufacturer´s protocol. qRT-PCR was performed using QuantiFast SYBR Green PCR Kit (QIAGEN, 10.3 ng of cDNA template, 500 nM primer, final volume 10 µl) for bovine samples and SYBR-Select Mastermix (Thermo Fisher, 10.3 ng template, 200 nM primer, final volume 10 µl) for human samples. The primer pair sequences of studied genes are listed in **Table 1**. Analysis was performed using the ΔΔct method. Bovine or human hypoxanthine guanine phosphoribosyl transferase (*Hprt1*) were used as the respective housekeeping genes.

**Table 1:** Primers used.

Number	Target	fwd/rev	Sequence 5’ -> 3’
a828	Bovine HPRT1	fwd	CTTTGCCGACCTGTTGGATTAC
a829	Bovine HPRT1	rev	CAATTACTTTTATGTCGCCTGTTGAC
a768	Bovine CD86	fwd	CAGGCTCGTATCAATGTTTCATCC
a769	Bovine CD86	rev	GCAATTAGTCTTATTTCTGGTTGACTG
a935	Bovine CD206	fwd	GGTGCCTCCAGTAAAACAAGC
a936	Bovine CD206	rev	TTGATACTAGCTAGATCTCCACCC
a975	Bovine IL10	fwd	GTGATGCCACAGGCTGAGAA
a976	Bovine IL10	rev	TGCTCTTGTTTTCGCAGGGCA
a977	Bovine TNFα	fwd	TCTTCTCAAGCCTCAAGTAACAAG
a978	Bovine TNFα	rev	CCATGAGGGCATTGGCATAC
a1378	Bovine IL6	fwd	AAGTGCACACCCGTCGTATT
a1379	Bovine IL6	rev	TCAGATTCAAGGCTGCTGGG
a1360	Bovine Socs3	fwd	TGAACGCAGTGCGCAAGCT
a1361	Bovine Socs3	rev	TGGGTCTTGACGCTGAGGGT
a1374	Bovine PIAS3	fwd	CGCCTGCGATGTCTCAAGATGG
a1375	Bovine PIAS3	rev	GCTTCCGTCCACTCTTGTTCCG
a927	Human HPRT1	fwd	GACCTGCTGGATTACATCAAAGC
a928	Human HPRT1	rev	GTCCCCTGTTGACTGGTCATT
a899	Human CD86	fwd	CTGTATTCTGGAAACTGACAAGACG
a900	Human CD86	rev	CTGTTGGAAGTACAGCTGTAATCC
a917	Human CD206	fwd	CTGGGTGGAGACTTAGCTAGC
a918	Human CD206	rev	GAAGGGCTTCCATATGTCAATCC
a923	Human IL10	fwd	CCAGACATCAAGGCGCATG
a924	Human IL10	rev	GTTTTCACAGGGAAGAAATCGATG
a921	Human TNFα	fwd	AACCCCGAGTGACAAGCC
a922	Human TNFα	rev	TGGTTATCTCTCAGCTCCACG
a1348	Human IL6	fwd	CAGGAGCCCAGCTATGAACTCCT
a1349	Human IL6	rev	GCGGCTACATCTTTGGAATCTTCTCC
a1354	Human Socs3	fwd	CCCAAGGACGGAGACTTCGATTC
a1355	Human Socs3	rev	GGGAAACTTGCTGTGGGTGACC
a1372	Human PIAS3	fwd	TCATCAGATGAGGAGGATCTGCCC
a1373	Human PIAS3	rev	CATAGCAGGGCTCCTTAGCACC

### Immunoblotting

Proteins were separated by SDS-PAGE Bis-Tris gradient gel (Thermo Fisher) and transferred to a PVDF membrane (Millipore). Proteins were detected with antibodies against HIF1α (Biomol), actin (Merck), pSTAT3 and STAT3 (Cell Signaling), respectively. Detection was performed using horseradish peroxidase conjugated secondary antibodies (Dianova) and a chemiluminescence detection system. Densitometry was performed with ImageJ (NIH).

### Indirect immunofluorescence

Cells were seeded on coverslips in 24-well cell plates. One hour prior to fixation, Lysotracker red (Thermo Fisher Scientific) was added (1:1000 final conc.). The cells were fixed with 4% paraformaldehyde (Alfa Aesar) in PBS (Merck) and permeabilized with ice-cold methanol before quenching with PBS containing 5% goat serum and 50mM NH_4_Cl. Next, the cells were incubated with an anti-*C. burnetii* antibody (rabbit, in house) and an anti-rabbit antibody labeled with AlexaFluor^®^488 Dye (Dianova). The samples were mounted onto glass slides using ProLong™ Diamond Antifade Mountant with DAPI (Thermo Fisher) to stain DNA. Sample analysis was performed using a Zeiss Apotome or a Zeiss LSM 700 confocal microscope.

### Statistical analysis

The statistical analysis was performed using GraphPad Prism 9. As stated in the figure legends, a one-sample t-test (if datasets are compared to normalized values), a paired or unpaired t-test (for normally distributed datasets), a Mann-Whitney test (for non-normally distributed datasets) or a two-way ANOVA with Tukey’s multiple comparison test were used. A value of p < 0.05 was considered significant.

## Results

### *C. burnetii* establishes a replicative phagolysosomal-like compartment in bovine lung epithelial cells

**Figure 1:**
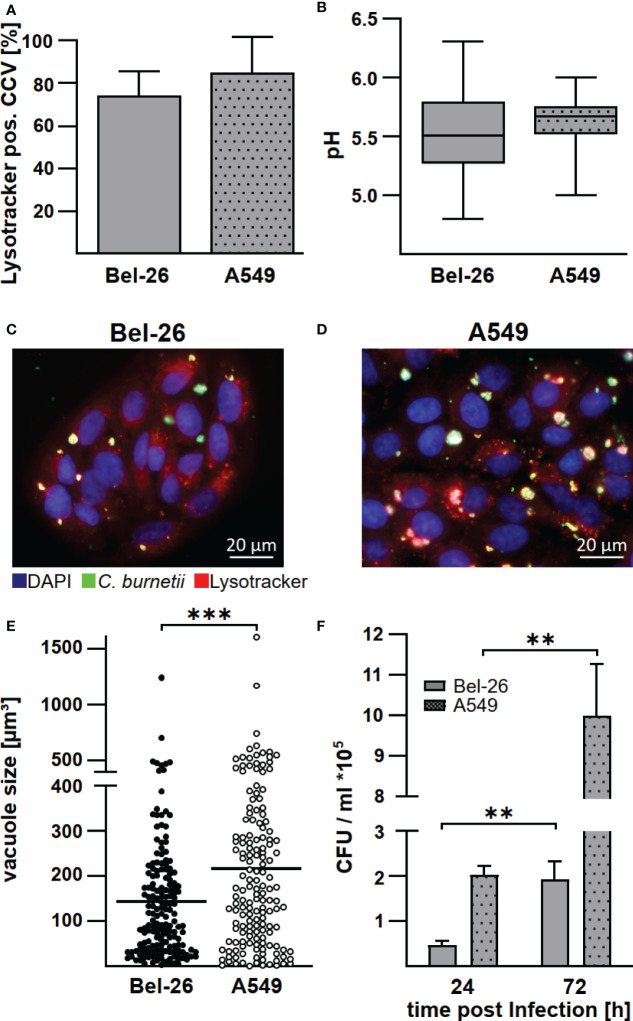
*C. burnetii* replicate in bovine and human lung epithelial cell lines in a lysosome-like compartment. The bovine lung epithelial cell line Bel-26 and the human lung epithelial cell line A549 were infected with *C*. *burnetii* NMII. **(A)** At 47 h post-infection, 1µM LysoTracker Red DND-99 was added to the culture medium for 1h. The cells were fixed, permeabilized and stained with an anti-*C. burnetii* antibody and DAPI. The percentage of LysoTracker positive *C. burnetii*-containing vacuoles (CCVs) of 100 infected cells in each of three independent experiments was determined. Mean ± SD. **(B)** The pH of the CCVs at 48 h post-infection was determined by calculation of the fluorescence intensity ratios of the dual-wavelength fluorophore LysoSensor Yellow/Blue DND-160. Data represents average values ± SD of 50 infected cells per sample from three independent experiments. **(C, D)** Representative immunofluorescence micrographs of **(C)** Bel-26 and **(D)** A549 cells infected with *C. burnetii* NMII for 48 h using a ApoTome (Zeiss). **(E)** The dimension of CCVs at 72 h post-infection was determined from confocal Z-stack images using the the LSM700 microscope and Zen (Zeiss) software. The volume of at least 100 CCVs from two independent experiments are shown. An unpaired t-test was performed. ***p < 0.001. **(F)** *C. burnetii* counts of either infected Bel-26 or A549 cells were determined at the time points indicated *via* counting of colony forming units (CFU). Shown is a representative experiment out of three experiments with similar results, performed with technical triplicates of biological duplicates. Mean ± SD, n = 6, Mann-Whitney test. **p<0.01.

Macrophages are the primary target cells of *C. burnetii* (1), but lung epithelial cells are the first contact of *C. burnetii* upon entering the host organism. There is evidence that bovine lung epithelial cells, being less susceptible to *C. burnetii* infection, do not act as replication sites (17). However, the infection characteristics have not been compared to a *C. burnetii-*infection in human lung epithelial cells. Thus, we infected the bovine and human lung epithelial cell lines Bel-26 and A549, respectively, with *C. burnetii* and determined the efficiency of invasion, replication ability, pH and characteristic properties of the *C. burnetii*-containing vacuole (CCV). First, we analyzed the MOI required to achieve an infection rate of ~70%. While in human A549 cells an MOI of 100 was necessary to reach an infection rate of ~70% at 48 h post-infection, an MOI of 200 was required in bovine Bel-26 cells (data not shown). In both cell lines, *C. burnetii* was able to establish LysoTracker-positive CCVs (**Figure 1A**). The pH of the CCVs in these two cell lines did not differ and was slightly acidic with pH values of 5.5 - 5.6 (**Figure 1B**). At 48 h post-infection, the average number of CCVs per cell was similar with 1.3 in Bel-26 and 1.6 in A549 cells (**Figures 1C, D**). In contrast to Bel-26 cells, A549 cells harbored an increased percentage of larger CCVs (**Figure 1E**) and higher bacterial counts (**Figure 1F**). Thus, at 24 h post-infection A549 cells harbored more than twice as many *C. burnetii* than Bel-26 cells (**Figure 1F**). However, *C. burnetii* replicated similarly well in both cell lines, and multiplied 4 - 5 fold from 24 to 72 h. This data indicated that, notwithstanding the differences detected in invasion efficiency, *C. burnetii* had established a replicative, LysoTracker-positive compartment in both human and bovine epithelial lung cells and species differences were minor at this level. Whether these pattern reflect the host species or just the respective properties of the cell lines used is unknown. Therefore, we focused on the analysis of the interaction of *C. burnetii* with primary cells and compared primary peripheral blood derived macrophages from humans and cattle under conditions present at the respiratory mucosal surface (normoxia) and in infected and inflamed tissue (hypoxia).

### *C. burnetii* replicated under hypoxia only in bovine, but not in human macrophages

**Figure 2:**
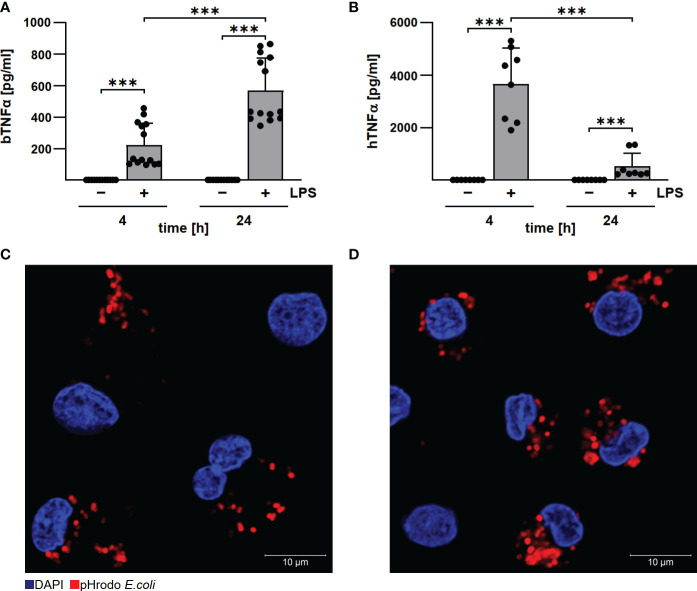
Human and bovine macrophages react to LPS and have phagocytic capacity. Bovine **(A)** and human **(B)** macrophages were stimulated with 10 µg/ml LPS for the time periods indicated. Cells were incubated for 4 and 24 h under normoxia. TNFα levels were analyzed by ELISA. Mean ± SD, n = 3, two-way ANOVA with Tukey’s multiple comparisons test. ***p<0.001. **(C, D)** Bovine and human macrophages were incubated with pHrodo Red *E. coli* bioparticles for 30 min. The cells were stained with DAPI. Representative immunofluorescence micrographs of **(C)** bovine and **(D)** human macrophages using a confocal laser scanning microscope (LSM700) are shown.

Established protocols for generating differentiated macrophages from human and bovine blood (19, 22) differ in procedure and cultivation media. To ensure comparability, we first analyzed basic functions of the differentiated macrophages. Bovine and human macrophages secreted TNFα after LPS stimulation, although with different kinetics (**Figures 2A, B**), and phagocytosed pHrhodo Red *E.coli* similarly well (**Figures 2C, D**). This data indicate that the bovine and human primary macrophage cultures used herein display a similar level of differentiation.

**Figure 3:**
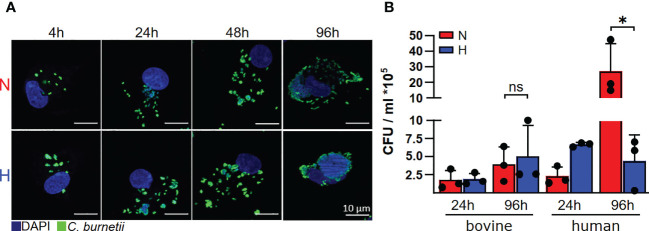
*C. burnetii* replication is inhibited by human, but not by bovine macrophages. **(A)** Bovine macrophages were infected with *C. burnetii* NMII for the time periods indicated under normoxic (N) or hypoxic (H) conditions. The cells were fixed and stained with DAPI and an anti-*C. burnetii* antibody. Representative immunofluorescence micrographs from three independent experiments with similar results are shown. **(B)** Human macrophages from three different donors and three independent bovine macrophage preparations from two bovine donor animals were infected with *C. burnetii* NMII for 24 and 96 hours under normoxic (N) or hypoxic (H) conditions. Bacterial counts were determined by counting colony forming units (CFU). Unpaired t-test or Mann-Whitney test, n=3. *p<0.05, ns=p>0.05.

Since we had discovered recently, that *C. burnetii* replicated only in the presence of oxygen in human and murine primary macrophages (22), we analyzed the influence of oxygen availability on the ability of *C. burnetii* to replicate intracellularly in bovine macrophages. In contrast to human macrophages, *C. burnetii* replicated in bovine macrophages under both normoxic and hypoxic conditions. This was demonstrated by immunofluorescence imaging of infected cells (**Figure 3A**) and by colony forming unit (CFU) analysis (**Figure 3B**), implying that the host cell species might be decisive for *C. burnetii* replication under hypoxic conditions.

### *C. burnetii*-induced modulation of the HIF1α-STAT3 axis differs between human and bovine macrophages

**Figure 4:**
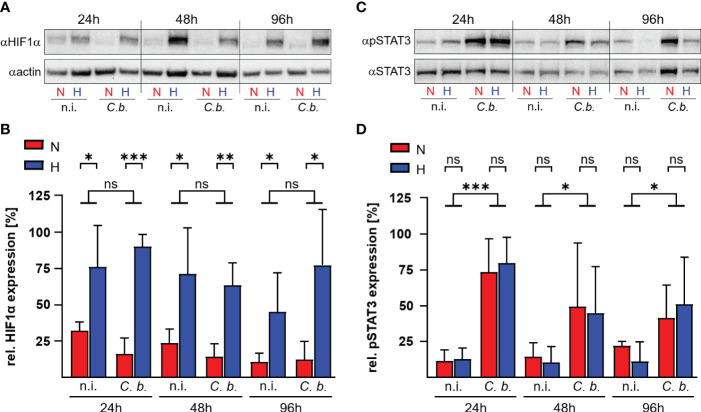
*C. burnetii* infection induces STAT3 activation in hypoxic bovine macrophages. Bovine monocyte derived macrophages were infected (*C. b.*) or not infected (n.i.) with *C*. *burnetii* NMII for the time periods indicated and kept under normoxic (N) or hypoxic (H) conditions. **(A, B)** The samples were subjected to immunoblot analysis using antibodies against HIF1α or actin. **(A)** One representative immunoblot of three independent experiments with similar results is shown. **(B)** The ratio of HIF1α to actin expression was calculated using ImageJ. Mean ± SD, n = 3, student´s t-test. ***p<0.001, **p*<*0.01, *p*<*0.05, ns>0.05. **(C, D)** The samples were subjected to immunoblot analysis using antibodies against phospho-STAT3 (pSTAT3) or STAT3. **(C)** One representative immunoblot of three independent experiments is shown. **(D)** The ratio of pSTAT3 to STAT3 expression was calculated from the immunoblots using ImageJ. Mean ± SD, n = 3, unpaired t-test. ***p<0.001, *p<0.05, ns>0.05.

HIF1α is the key transcription factor allowing the cell to adapt and to react to shifts in oxygen content (29). Under ample oxygen availability, proline residues 402 and 564 of HIF1α are hydroxylated by prolyl hydroxylases (PHD), which are cellular oxygen sensors. Hydroxylated HIF1α is recognized by the ubiquitin ligase Von Hippel-Lindau protein (pVHL), which marks HIF1α for proteasomal degradation (30, 31). When oxygen is missing, the PHDs are disabled, which leads to stabilization of HIF1α and, as a consequence, to the expression of genes involved in the adaption to hypoxia, metabolic processes and immune system regulation (23, 32). HIF1α also prevents STAT3 activation, thereby interfering with *C. burnetii* replication inside hypoxic human macrophages (22). Therefore, we analyzed the HIF1α protein levels in bovine macrophages infected with *C. burnetii* under normoxic and hypoxic conditions. Hypoxia induced stabilization of HIF1α, which was not further enhanced by *C. burnetii* infection (**Figures 4A, B**). This is in contrast to our observation in human and murine macrophages, where the infection resulted in augmented HIF1α levels, which impaired the activation of STAT3, resulting in inhibition of *C. burnetii* replication (22). Interestingly, *C. burnetii* infection of bovine macrophages resulted in phosphorylation of STAT3 independent of the available oxygen concentration (**Figures 4C, D**). This data suggests that the activation of STAT3 under hypoxia seems to be distinct in different *C. burnetii* host-species. In human and murine macrophages, hypoxia leads to stabilization of HIF1α, to inhibition of STAT3 activation, and inability of *C. burnetii* to replicate (22). In hypoxic bovine macrophages, in contrast, STAT3 was activated and *C. burnetii* multiplied.

### STAT3 regulators differ in their expression level in *C. burnetii*-infected bovine and human macrophages

**Figure 5:**
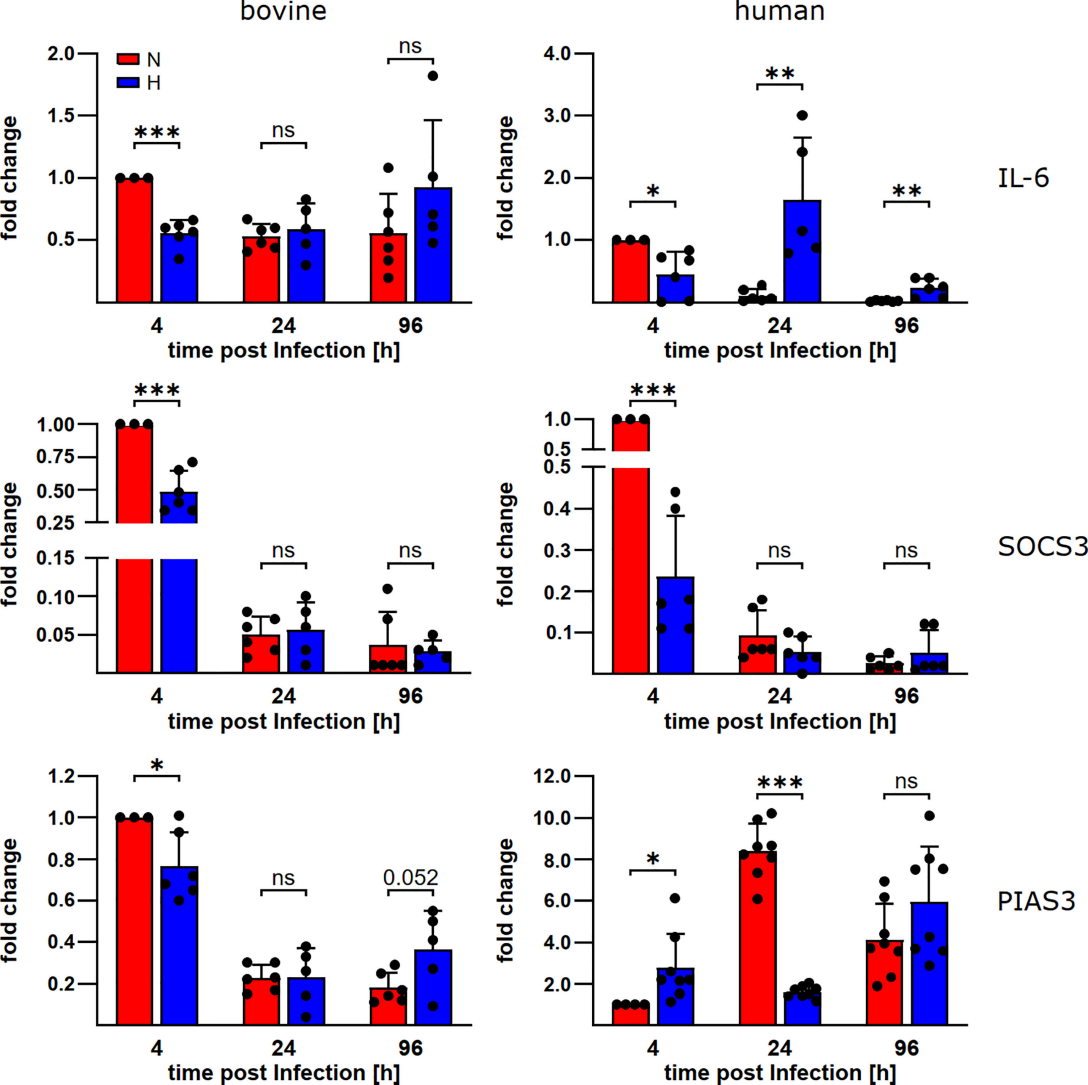
*C. burnetii*-infection influences gene expression of STAT3 regulators. Gene expression was determined for the genes encoding IL-6, SOCS3 and PIAS3 in *C. burnetii* NMII infected normoxic and hypoxic bovine (left) and human (right) macrophages at the time points indicated. The data from three to four independent experiments are shown as mean ± SD of 2^-ΔΔCT values (using human or bovine HPRT as a calibrator). Fold changes are shown relative to the 4 h time point under N. One-sample t-test, unpaired t-test or Mann-Whitney test, n=5-6 (bovine) and n=6-8 (human) ***p<0.001, **p<0.01, *p<0.05, ns=p>0.05.

To learn why STAT3 is activated in *C. burnetii*-infected bovine macrophages, but not in *C. burnetii*-infected human macrophages under hypoxia, we analyzed the mRNA levels of IL-6, a known STAT3 inducer, and SOCS3 and PIAS3, two known STAT3 inhibitors (33). We detected differences in the expression levels of IL-6 and PIAS3 during *C. burnetii*-infection between bovine and human macrophages (**Figure 5**). In bovine macrophages, oxygen availability neither influenced the expression level of IL-6 nor that of the at 24 and 96 h post-infection. In contrast, the IL-6 expression level was increased in human macrophages under hypoxia at 24 and 96 h post-infection and the PIAS3 expression level was increased at 24 h post-infection under normoxia. However, these expression patterns only partially explain the STAT3 activation levels described above (**Figures 4C, D**). The observed pattern of STAT3 inducer and inhibitor expression apparently is not compliant with mitigated STAT3 activation in hypoxic human macrophages. Interestingly, we observed an oxygen-independent expression of the STAT3 activator IL-6, while the STAT3 inhibitors PIAS3 and SOCS3 were strongly reduced in *C. burnetii*-infected bovine macrophage (**Figure 5**), which might favor activation of STAT3 under both, normoxic and hypoxic conditions in bovine macrophages.

### *C. burnetii*-induced modulation of the level of TCA metabolites differs between human and bovine macrophages

**Figure 6:**
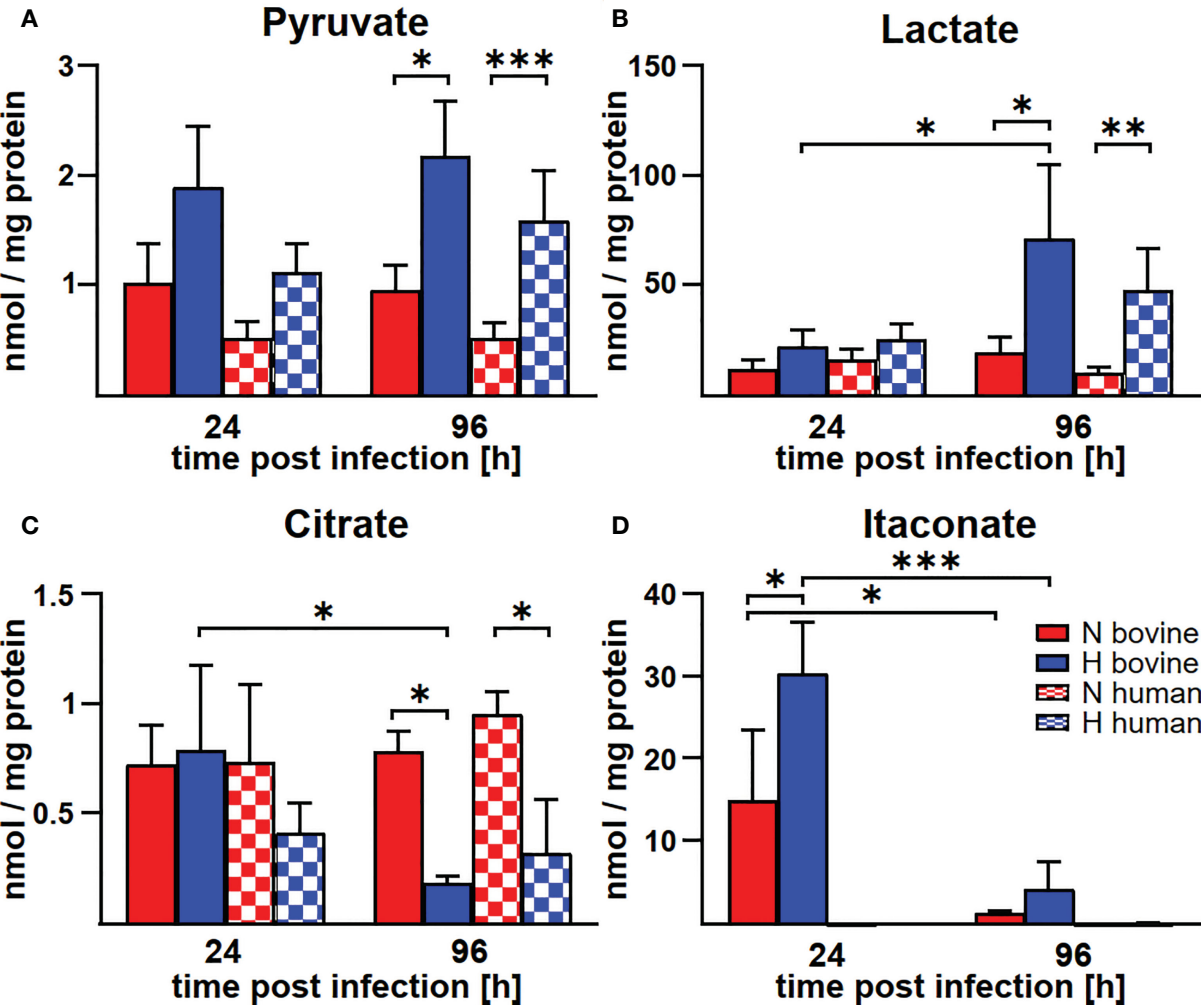
Hypoxia alters metabolite concentrations in *C. burnetii*-infected human and bovine macrophages similarly, with the exception of itaconate. **(A-D)** Human and bovine macrophages were infected with *C. burnetii* NMII for the time periods indicated under normoxia (N) or hypoxia (H). Carboxylic acids were analyzed by GC-MS. The amounts of **(A)** pyruvate, **(B)** lactate, **(C)** citrate, and **(D)** itaconate respectively, are shown in nmol/mg protein from three to four independent experiments. Mean ± SD, n = 3-4, two-way ANOVA with Tukey’s multiple comparison test. ***p<0.001, **p<0.01, *p<0.05.

**Figure 7:**
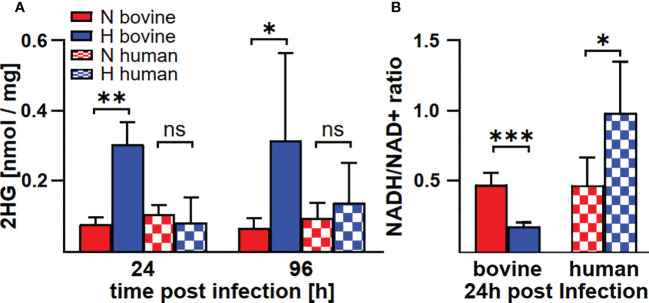
*C. burnetii* infection induces an increase of 2HG and a reduction of the NADH/NAD+ ratio only in hypoxic macrophages. Human and bovine macrophages were infected with *C. burnetii* NMII for the time periods indicated under normoxia (N) or hypoxia (H). **(A)** The amounts of 2HG (2-hydroxyglutarate) were analyzed by GC-MS and are shown in nmol/mg protein for three to four independent experiments. Unpaired t-test, n=3-4. **p<0.01, *p<0.05, ns=p>0.05. **(B)** NADH and NAD+ were analyzed in pmol using a colorimetric NAD+/NADH assay. The NADH/NAD+ ratio was calculated from 2-3 independent experiments performed in duplicate or triplicate. Mean ± SD, unpaired t-test. ***p<0.001, **p<0.01, *p<0.05.

In human and murine macrophages, hypoxia leads to a limitation of citrate, which results in the inability of *C. burnetii* to replicate (22). Thus, we assessed the carboxylic acid metabolism in *C. burnetii*-infected hypoxic bovine macrophages to determine its potential role in the ability of *C. burnetii* to replicate. The levels of pyruvate and lactate were generally higher in infected hypoxic than normoxic human and bovine macrophages, but only the level of lactate showed a significant increase in hypoxic human and bovine macrophages over the time course of infection (**Figures 6A, B**). Under hypoxic conditions, we observed reduced citrate levels in *C. burnetii*-infected human macrophages (**Figure 6C**), similar to previous results in *C. burnetii*-infected murine macrophages (22). Although we did not observe citrate level reduction in *C. burnetii*-infected hypoxic bovine macrophages at 24 h post-infection, it was similarly reduced under hypoxia as in human macrophages at 96 h post-infection (**Figure 6C**). This indicates that the level of citrate under hypoxia might not be responsible for the ability of *C. burnetii* to replicate in hypoxic bovine macrophages. Furthermore, *C. burnetii*-infected human macrophages hardly produced itaconate under all conditions tested (**Figure 6D**), which is in line with previous observations (34). In contrast, infected bovine macrophages produced significant amounts of itaconate, with higher levels under hypoxic conditions. These levels decreased during the infection under both conditions (**Figure 6D**). Itaconate is derived from citrate, can prevent growth of several bacteria and has immune-regulatory function (34–36). As itaconate also inhibits growth of *C. burnetii* (37), the increased level of itaconate in bovine macrophages contrasts with the ability of *C. burnetii* to replicate intracellularly. Another metabolite, whose intracellular concentration was significantly increased under hypoxic conditions in bovine, but not human *C. burnetii*-infected macrophages was 2-hydroxyglutarate (2HG) (**Figure 7A**). An acidic intracellular environment resulting from oxygen limitation is believed to favor the reduction of glutamine-derived α-ketoglutarate to 2HG by lactate dehydrogenase A and malate dehydrogenase, with the increase in 2HG varying substantially among different cell types from about 2- to 25-fold (38). Production of intracellular 2HG consumes NADH, thereby alleviating hypoxia-induced reductive stress, which is defined as an excess accumulation of reducing equivalents brought about mostly by suppressed oxidation of NADH by respiratory complex I under hypoxia. In line, we found the NADH-to-NAD^+^ ratio, to be lower in hypoxic bovine macrophages, but not in hypoxic human macrophages at 24 h post-infection (**Figure 7B**).

Neither a *C. burnetii* infection nor the level of available oxygen affected the intracellular levels of the Krebs cycle intermediates succinate, fumarate, and malate in both human and bovine macrophages (**Table 2**).

**Table 2:** Krebs cycle intermediates under normoxia and hypoxia.

			Normoxia		Hypoxia		
Metabolite	Time	Species	Average	StDev	Average	StDev	*p value*
Succinate	24h	Bovine	0.47	0.17	0.74	0.14	0.095
		Human	0.53	0.21	0.26	0.11	0.059
	96h	Bovine	0.20	0.05	0.25	0.12	0.474
		Human	0.34	0.07	0.22	0.17	0.241
Fumarate	24h	Bovine	0.54	0.28	0.59	0.22	0.835
		Human	0.33	0.10	0.33	0.05	0.957
	96h	Bovine	0.68	0.22	0.41	0.27	0.171
		Human	0.29	0.07	0.34	0.34	0.756
Malate	24h	Bovine	0.93	0.18	0.89	0.37	0.872
		Human	0.72	0.08	0.72	0.26	0.997
	96h	Bovine	0.67	0.09	0.64	0.38	0.883
		Human	0.65	0.23	0.74	0.34	0.689
α-KG	24h	Bovine	0.66	0.76	1.02	0.96	0.640
		Human	0.22	0.12	0.11	0.05	0.130
	96h	Bovine	0.74	0.17	0.24	0.16	0.004
		Human	0.71	0.60	0.35	0.30	0.320
2HG	24h	Bovine	0.08	0.02	0.31	0.06	0.004
		Human	0.11	0.03	0.08	0.07	0.574
	96h	Bovine	0.07	0.03	0.32	0.25	0.093
		Human	0.10	0.04	0.14	0.11	0.507
Glucose	24h	Bovine	14.54	9.14	11.17	8.41	0.662
		Human	1.84	0.90	2.87	1.01	0.180
	96h	Bovine	11.51	1.31	2.78	1.77	0.000
		Human	0.71	0.34	1.43	0.44	0.042

Human and bovine macrophages were infected with *C. burnetii* NMII for the time periods indicated, under normoxia or hypoxia. Metabolites were analyzed by GC-MS. The amount of each metabolite is shown in nmol/mg protein from three to four independent experiments. α-KG (alpha ketoglutarate). Mean ± SD, n = 3-4, unpaired t-test.

### *C. burnetii*-induced upregulation of TNFα expression was stabilized only under hypoxia in human macrophages

**Figure 8:**
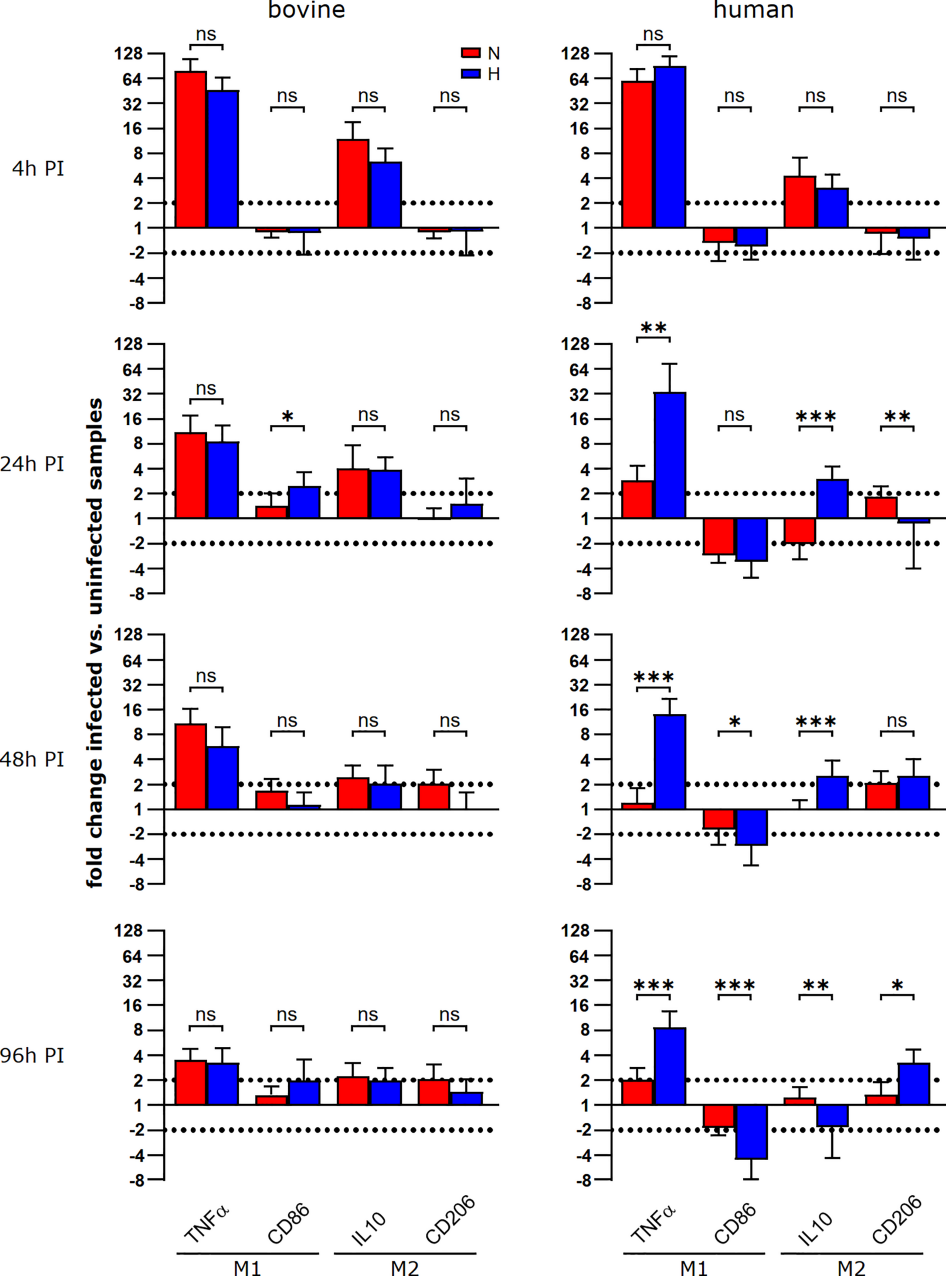
During the course of a *C. burnetii* infection, TNFα expression is only maintained in hypoxic human macrophages. Gene expression was determined for M1/M2 polarization marker genes in *C. burnetii* NMII infected human or bovine macrophages at the time points indicated. Data are shown as mean ± SD of ΔΔCT values (using uninfected samples as a calibrator), Mann-Whitney test, n=5-8 (bovine) and n=6-10 (human) ***p<0.001, **p<0.01, *p<0.05, ns=p>0.05.

To investigate why *C. burnetii* replication was supported in hypoxic bovine macrophages, but not in hypoxic human macrophages, we analyzed the respective macrophage polarization, as the infection with *C. burnetii* stimulates an atypical M2 phenotype in human macrophages (39). Indeed, a similar polarization of murine macrophages towards an M2 phenotype is important for the permissiveness of these host cells to *C. burnetii* replication (40). Thus, we evaluated the expression of two M1 genes (TNFα and CD86) and two M2 genes (IL-10 and CD206) at different time points post-infection under normoxic and hypoxic conditions in human and bovine macrophages. In human macrophages, we observed that the infection induced a strong upregulation of TNFα and a slight upregulation of IL-10 at 4 h post-infection independent of the availability of oxygen (**Figure 8**). Over the time-course of the infection, the induction of the genes analyzed decreased gradually. Importantly, the levels of TNFα mRNA at 24 and 96 h post-infection were significantly higher in infected human macrophages kept under hypoxia. *C. burnetii*-infected bovine macrophages were also characterized by an upregulation of TNFα and IL-10 at early time-points post-infection. Like in infected human macrophages, the upregulation of the genes analyzed decreased over time. In contrast to the situation in human cells, we did not observe a difference in TNFα induction between infected hypoxic and normoxic bovine macrophages (**Figure 8**). As TNFα is a cytokine known to restrict *C. burnetii* replication (41), the difference in TNFα expression might explain why *C. burnetii* is capable of replicating in hypoxic bovine macrophages, but not in hypoxic human macrophages.

### Neither normoxic nor hypoxic *C. burnetii*-infected bovine macrophages secrete TNF

**Figure 9:**
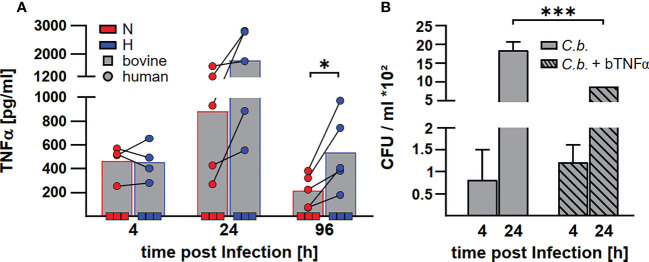
*C. burnetii* replication in infected bovine macrophages correlates with the lack of TNFα secretion. **(A)** Human macrophages from four different donors (circle) and three independent bovine macrophage preparations from two bovine donor animals (square) were infected with *C. burnetii* NMII for 4, 24 and 96 h under normoxia (N) or hypoxia (H). TNFα levels were analyzed by ELISA. Mean ± SD, paired t-test. *p<0.05. **(B)** Three independent bovine macrophage preparations from two bovine donor animals were infected with *C. burnetii* NMII and either left untreated or treated with 10ng/ml bTNFα. Cells were incubated for 4 and 24 h under normoxia. Bacterial counts were determined by counting colony forming units (CFU). Shown is a representative experiment performed with technical triplicates out of two experiments with similar results. Mean ± SD, unpaired t-test. ***p<0.01, *p<0.05.

Levels of transcription of cytokine genes and of secretion of the encoded proteins do not necessarily correlate (42). In addition, not only the expression, but also the release of TNFα is controlled by HIF1α (24). In the supernatant of *C. burnetii* infected human macrophages, we observed the highest amount of TNFα at 24 h post-infection. The level of TNFα decreased over time. At later time points post-infection, the amount of TNFα in the supernatant was higher for infected hypoxic than normoxic human macrophages (**Figure 9A**), confirming that the cytokine levels correlated with the mRNA levels (**Figure 8**). Although bovine macrophages proved capable of secreting bTNFα after LPS stimulation (**Figure 2A**), we could not detect bTNFα in the supernatant of *C. burnetii*-infected bovine macrophages under all conditions tested using either a bTNFα-specific ELISA (**Figure 9A**) or a TNFα bioassay (not shown).

### TNF is involved in the control of *C. burnetii* replication in bovine macrophages

As the lack of TNFα secretion might contribute to the lack of control of *C. burnetii* replication in hypoxic bovine macrophages (41), we treated *C. burnetii*-infected bovine macrophages with bTNFα and analyzed CFU counts at different time points post-infection under normoxic conditions. Addition of bTNFα to *C. burnetii*-infected bovine macrophages resulted in lower bacterial counts (**Figure 9B**), suggesting that the lack of TNFα in *C. burnetii* infected bovine macrophages is important for the inability to prevent bacterial replication under hypoxic conditions.

## Discussion

Once *C. burnetii* is internalized, the *C. burnetii*-containing vacuole (CCV) matures to an acidic, phagolysosomal-like parasitophorous vacuole (27, 43). During the course of the cellular infection, early phagolysosomes harbor a single bacterial cell. Sequentially, they fuse to a single large parasitophorous vacuole containing multiple *C. burnetii* cells (44). Mature CCVs have a pH of 4.7 to 5.2, which stimulates the activation of bacterial metabolism and the assimilation of essential nutrients (45–47). *C. burnetii* requires a functional Dot/Icm type IV secretion system (T4SS) for establishing the replicative CCV (48, 49). In addition to pH tolerance, a dismutase-catalase system promotes intracellular survival of *C. burnetii*. It eliminates reactive oxygen species generated by the bacteria themselves but also, most importantly, those generated by the host cell (50). Consequently, the infectious process might differ at multiple points/steps that are decisive for host differences in *C. burnetii* pathogenesis.

Previous studies have shown that species differences do exist down to the cellular level, however, genetic background and/or molecular basis have not been uncovered (19). Hypoxia plays a major role in the host - C*. burnetii* interaction. Therefore, we analyzed the interaction of human and bovine monocyte-derived macrophages with *C. burnetii* under normoxic and hypoxic conditions. The following reasons prompted us to analyze the influence of hypoxia during the course of a *C. burnetii* infection: i) the oxygen level is low at the site of infection in an acute colitis model (51); ii) murine macrophages prevent *C. burnetii* replication under hypoxia; iii) *C. burnetii* might enter a state of persistence under hypoxia (22); and iv) the course of a *C. burnetii* infection differs in humans and cattle (1). Our results indicate that the host-pathogen interaction differs also at the cellular level. Thus, *C. burnetii* replication is controlled in hypoxic human monocyte-derived macrophages, but not in bovine monocyte-derived macrophages (**Figure 3**). This difference might be of biological importance. In hypoxic human macrophages, *C. burnetii* might enter a stage of persistence, similar to the situation in hypoxic murine macrophages (22). Thus, hypoxia and/or HIF1α prevent bacterial replication without elimination of the pathogen (52), which might allow reoccurring or chronic infections. In the case of *C. burnetii*, it might support and/or allow Q fever to reach a chronic state in humans. Interestingly, infection with *C. burnetii* is common in cattle, but clinical disease is rare (53). Infertility, abortion and mastitis have been reported (54). However, there are conflicting reports whether seropositive cows have better or worse reproduction (54, 55). Whether the difference in controlling *C. burnetii* infection under hypoxia might result in different clinical outcomes can only be speculated about. Importantly, the lack of control of *C. burnetii* replication by hypoxic bovine macrophages might be mediated by STAT3 activation (**Figure 4**), as *C. burnetii* replication in murine macrophages under normoxia depends on the presence of STAT3. In addition, expression of a constitutively active STAT3 in murine macrophages also allows replication under hypoxia (22). The observed constant expression of the STAT3 activator IL-6 along with strongly limited expression of the STAT3 inhibitors PIAS3 and SOCS3 in *C. burnetii*-infected bovine macrophage (**Figure 5**) could favor activation of STAT3 and might explain the deficit of bovine macrophages to control *C. burnetii* replication under oxygen-limited conditions. However, the pathway(s) leading to STAT3 activation in *C. burnetii*-infected hypoxic bovine macrophages and how this impacts *C. burnetii* replication has to be analyzed in more detail.

The transcription factor STAT3 has key roles in inflammation and immunity (33). It suppresses signal transduction mediated by TLRs (56) and has anti-inflammatory function in mice and in humans (33). In addition, STAT3 also affects cellular metabolism. Thus, STAT3 increases the expression of Indy, a citrate transporter, and of citrate synthase, thereby elevating the intracellular level of the TCA metabolite citrate (57, 58). Indeed, higher citrate levels were only observed in *C. burnetii*-infected normoxic, but not hypoxic human macrophages (**Figure 6C**), suggesting that the increased level of STAT3 correlates with increased citrate levels (**Figures 4**, **6C**). However, we were unable to observe this correlation in bovine macrophages at 96 h post-infection (**Figures 4**, **6C**). Whether this might point to a difference in regulation of host cell metabolites between bovine and human macrophages has to be clarified. The significant difference between the cellular levels of itaconate supports such an assumption. Itaconate is produced by IRG1 from cis-aconitate, which is produced by ACO2 from citrate (59). We did not detect itaconate in *C. burnetii*-infected human macrophages (**Figure 6D**), in agreement with previous observations (34). In contrast, normoxic murine macrophages infected with *C. burnetii* and *Legionella pneumophila* produced high amounts of itaconate, which was reduced under hypoxic conditions (22). *C. burnetii* infected bovine macrophages also produced itaconate, but, in contrast to murine macrophages, it was elevated under hypoxic conditions (**Figure 6D**). As itaconate impedes bacterial replication (36), it is unlikely that the increased itaconate level in infected hypoxic bovine macrophages accounts for *C. burnetii* replication. However, itaconate also has an immune-regulatory function. Thus, treatment of LPS-stimulated murine macrophages with itaconate or its derivatives reduced the production of the pro-inflammatory cytokines IL-1β and IL-6, but not of TNF (35, 60). As TNFα is essential in restricting *C. burnetii* in murine macrophages, increased itaconate levels might not permit *C. burnetii* replication. However, we observed a host species-specific difference in TNFα mRNA levels during infection. In infected human macrophages, TNFα expression was always higher under hypoxic conditions at all time-points tested, while in infected bovine macrophages we did not observe an influence of the oxygen level on the TNFα mRNA level (**Figure 8**). Importantly, bovine macrophages were herein found not to secrete TNFα in the context of *C. burnetii* infection, despite of infection-induced mRNA synthesis (**Figure 8** and **Figure 9**) and the ability to secrete TNFα after LPS stimulation (**Figure 2**). Thus, the ability of *C. burnetii* to replicate in hypoxic bovine macrophages might be mediated by a block of TNFα secretion. TNF trafficking and release to the extracellular milieu is well studied (61). After translation in the ER as type II membrane precursor pro-TNF, it is rapidly delivered to the Golgi, from where it traffics to the cell surface. The transport of TNF from the trans-Golgi network (TGN) depends on golgin and golgin-245 (62). TNF-loaded vesicles from the TGN fuse with recycling endosomes (RE), which depends on the SNARE proteins Stx6, Stx7 and Vti1b (63, 64). Transport of TNF from the RE to the cell surface is facilitated by Stx4 and VAMP-3 (63, 65). To be released as a soluble cytokine at the cell surface, pro-TNF has to be cleaved by the TNF-converting enzyme (TACE, ADAM17) (66). The block of TNFα secretion by *C. burnetii*-infected bovine macrophages may occur at any of these different steps. However, it might also be possible that *C. burnetii* activates or stabilizes synaptotagmin 11, which inhibits cytokine secretion (67). Further research will be required to elucidate at which point TNFα secretion is blocked and, more importantly, how.

**Figure 10:**
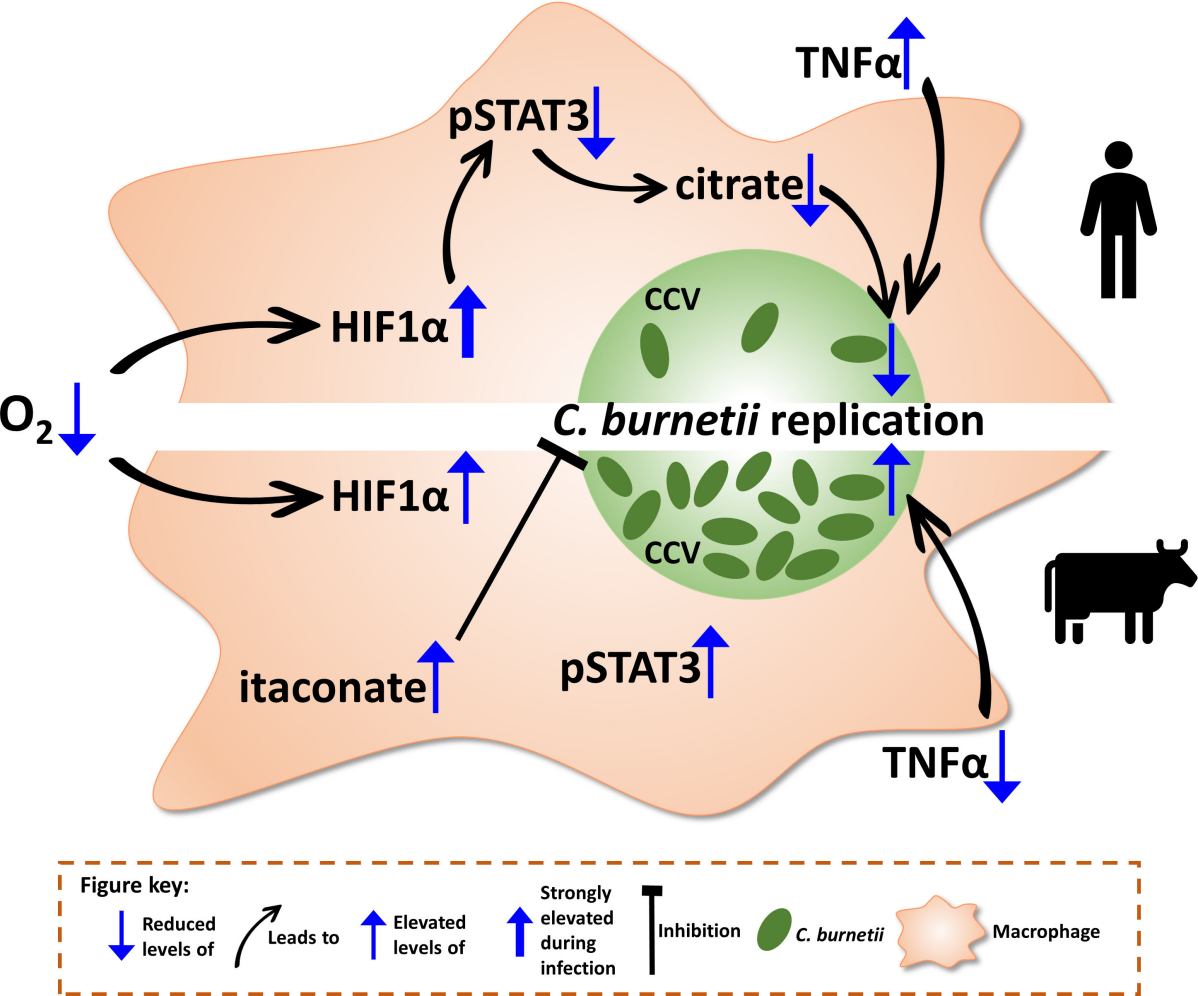
Schematic model of the consequences of hypoxia on bovine and human macrophages infected with *C. burnetii*. When oxygen (O_2_) levels drop, the transcription factor hypoxia-inducible factor 1-alpha (HIF1α) is stabilized. While in human macrophages (hMΦ), a *C. burnetii* infection leads to an elevation of HIF1α stabilization, HIF1α levels in bovine macrophages (bMΦ) are not altered upon infection. In infected hMΦ, HIF1α prevents activation of signal transducer and activator of transcription 3 (STAT3) by phosphorylation, which in turn causes a decrease in intracellular citrate levels, leading to the impediment of *C. burnetii* replication. However, this impairment of STAT3 activation by HIF1α is not observed in bMΦ causing phosphorylation of STAT3. Furthermore, the tumor necrosis factor alpha (TNFα) secretion is increased in hypoxic hMΦ correlating with the control of *C. burnetii* replication. In contrast, TNFα secretion is blocked in bMΦ, eliminating the factor of control over *C. burnetii*. Thus, *C. burnetii* is able to replicate. Finally, itaconate levels are increased in bMΦ, which have been shown to inhibit *C. burnetii* replication.

Taken together, our results indicate that *C. burnetii* activates distinct signaling cascades in human and bovine macrophages, leading to different levels of control of bacterial replication. The lack of control of *C. burnetii* by hypoxic bovine macrophages is associated with STAT3 activation and a block of TNFα secretion (**Figure 10**). Addition of TNFα enables the bovine macrophages to at least partially restrict bacterial replication (**Figure 9B**), supporting the importance of this cytokine for cell autonomous control of *C. burnetii* replication. It can only be speculated how this difference in controlling *C. burnetii* replication might contribute to disease severity. At first glance, it is counterintuitive that the lack of control might result in the development of less severe disease. However, if *C. burnetii* is controlled, but not eliminated, this might lead to persistence. Chronic Q fever develops months to years after primary infection (68), indicating that bacterial persistence is an important step in disease development. In contrast, constant low-level bacterial replication might activate the innate and adaptive immune systems, allowing better control of the pathogen *in vivo* and consequently prevention of disease.

## Data availability statement

The original contributions presented in the study are included in the article/supplementary material. Further inquiries can be directed to the corresponding authors.

## Ethics statement

The studies involving human participants were reviewed and approved by Ethical Committee Erlangen approval number 111_12B. Written informed consent for participation was not required for this study in accordance with the national legislation and the institutional requirements. The animal study was reviewed and approved by Permit numbers 22-2684-04-04-102/15 and 22-2684-04-BFI-20-102.

## Author contributions

AL conceived, designed and supervised the study. AL and CM provided resources. MM, MÖ, IH, JS-L, and KD performed the experiments. MM, IH, JS-L, KD, PO, CB, CM and AL analyzed the data. MM, IH, CB, PO, CM and AL wrote the manuscript. All authors contributed to the article and approved the submitted version.
